# Dihydromyricetin induces mouse hepatoma Hepal-6 cell apoptosis via the transforming growth factor-β pathway

**DOI:** 10.3892/mmr.2014.2891

**Published:** 2014-11-06

**Authors:** BIN LIU, WEI ZHOU, XIAOFENG CHEN, FENGMING XU, YINQIN CHEN, JIE LIU, QINGYU ZHANG, SHITING BAO, NIANPING CHEN, MINGYI LI, RUNZHI ZHU

**Affiliations:** 1 Laboratory of Hepatobiliary Surgery, Zhanjiang Key Laboratory of Hepatobiliary Diseases, Guangdong Medical College, Zhanjiang, Guangdong 524001, P.R. China; 2 Department of Pediatrics, Affiliated Hospital of Guangdong Medical College, Zhanjiang, Guangdong 524001, P.R. China; 3 Department of Interventional Medicine, Affiliated Hospital of Guangdong Medical College, Zhanjiang, Guangdong 524001, P.R. China

**Keywords:** dihydromyricetin, transforming growth factor-β, Smad3, NADPH oxidase 4, reactive oxygen species

## Abstract

Dihydromyricetin (DHM) is a flavonoid compound which possesses potent antitumor activity. In the present study, it was demonstrated that DHM significantly inhibited proliferation and induced apoptosis in mouse hepatocellular carcinoma Hepal-6 cells. Transforming growth factor β (TGF-β) is recognized as a major profibrogenic cytokine and is therefore a common target for drugs in the treatment of liver disease. The present study aimed to investigate whether TGF-β was involved in DHM-triggered cell-viability inhibition and apoptosis induction. An MTT assay was used to evaluate the viability of Hepal-6 cells following DHM treatment. TGF-β signalling is mediated by Smads and nicotinamide adenine dinucleotide phosphate oxidase 4 (NOX4) is a crucial regulator of reactive oxygen species ROS production. TGF-β, Smad3, phosphorylated (p)-Smad2/3 and NOX4 protein expression levels were evaluated by western blot analysis. TGF-β and NOX4 gene expression levels were determined by quantitative polymerase chain reaction. The results indicated that DHM downregulated TGF-β, Smad3, p-Smad2/3 and NOX4 in a concentration-dependent manner. A cell counting assay indicated that DHM also inhibited Hepal-6 cell growth in a concentration-dependent manner. TGF-β expression was significantly decreased following DHM treatment. In conclusion, the results of the present study defined and supported a novel function for DHM, indicating that it induced cell apoptosis by downregulating ROS production via the TGF-β/Smad3 signaling pathway in mouse hepatocellular carcinoma Hepal-6 cells.

## Introduction

Dihydromyricetin (DHM, C_15_H_12_O_8_, PubChem CID: 161557, Fig. 1A) is an active component in extracts of *Ampelopsis grossedentata* and a biologically active flavonoid compound (1). DHM possesses potent antitumor activity both *in vivo* and *in vitro* (2). It has been reported that DHM has numerous pharmacological functions, including anti-inflammatory, antibacterial, cough-relief, antioxidant, antihypertensive, hepatoprotective and anti-cancer effects (3,4). It exerts an antioxidative effect by chelating Fe^2+^ (5). In addition, it was demonstrated that DHM was able to decrease accumulation of reactive oxygen species (ROS) (6,7). Previous studies have reported significant inhibitory activity of DHM against breast cancer MCF-7 (8) and MDA-MB-231 (9) cells, nasopharyngeal carcinoma HK-1 cells, liver cancer Bel-7402 cells (10), leukemia HL-60 and K-562 cells and lung cancer H1299 cells (11). Based on evidence from previous studies, the present study aimed to elucidate the association between transforming growth factor-β (TGF-β) and nicotinamide adenine dinucleotide phosphate oxidase 4 (NOX4) during DHM-induced apoptosis in mouse hepatocellular carcinoma Hepal-6 cells.

Though TGF-β was initially suggested to be involved in a tumor supressor pathway due to its cytostatic activity in epithelial cells, further studies have identified TGF-β as a pro-tumorigenic factor. The majority of human tumors, including melanoma, secrete significant amounts of TGF-β, which directly influences the tumor microenvironment, promoting peritumoral angiogenesis as well as tumor cell migration and invasiveness, immune evasion and dissemination to metastatic sites (12,13). TGF-β signaling is mediated by TGF-type II (TβRII) and type I (TβRI) receptors. TGF-β binding induces the formation of heteromeric complexes which promote the phosphorylation, and therefore activation, of TβRI by TβRII. Activated TβRI phosphorylates receptor (R)-Smads, including Smad2 and -3 (14). These activated R-Smads form heteromeric complexes with Smad4, which accumulate in the nucleus and regulate target-gene transcription (15). TGF-β has been shown to increase NOX4 expression in various cell types; however, the localization of NOX4 remains to be elucidated (16). Tobar *et al* (17) reported that TGF-β upregulated NOX4 expression via a factor-induced apoptotic pathway in fetal rat hepatocytes. Furthermore, ROS production in human hepatocyte cell lines previously infected with the hepatitis C virus depends on NOX4 activity whose expression is stimulated by TGF-β (18). Several studies have reported that TGF-β promotes NOX4 production of intracellular ROS (19,20). ATP production and biosynthesis of building blocks are required to sustain cellular function and cell viability is functionally coordinated by interlocking regulatory mechanisms that control electron transport in the respiratory chain (21). The present study therefore aimed to investigate whether DHM was able to reduce ATP levels and ROS production via the TGF-β signaling pathway in mouse hepatoma Hepal-6 cells.

## Materials and methods

### Reagents

DHM was purchased from Sigma (St. Louis, MO, USA) and was dissolved to a concentration of 50 mM in dimethylsulfoxide (DMSO) as a stock solution and stored at −20°C. The final DMSO concentration did not exceed 0.1% DMSO throughout the study. Rabbit antibodies to TGF-β, TGF-βRII, Smad3, phosphorylated (p)-Smad2/3 and GAPDH were obtained from Cell Signaling Technology (Beverly, MA, USA). Goat anti-rabbit immunoglobulin G-horseradish peroxidase (IgG-HRP; EarthOx, Millbrae, CA, USA) was used as the secondary antibody.

### Cell culture and DHM treatment

The mouse Hepal-6 cell line was provided by the Maternal and Child Health Hospital of Shanghai (Shanghai, China). Cells were cultured in RPMI-1640 medium supplemented with 10% (v/v) fetal bovine serum (Gibco-BRL, Invitrogen Life Technologies, Carlsbad, CA, USA), penicillin 100 U/ml and streptomycin 100 U/ml (Hyclone, Logan, UT, USA), and maintained in a humidified atmosphere of 95% air and 5% CO_2_ at 37°C. Hepal-6 cells were grown in standard media and when the confluency reached 50–60%, cells were treated with DHM (10, 50 or 100 μM) for 48 h.

### Measurement of intracellular ROS levels

To detect the accumulation of intracellular ROS in Hepal-6 cells, a ROS assay kit was purchased from BioVision Inc. (Milpitas, CA, USA). Briefly, following treatment of cells with different concentrations of DHM (10, 50 and 100 μM) for 48 h in a 96-well plate at a cell density of 2500 cells/well, 100 μl 2′,7′-dichlorofluorescin diacetate (DCFDA) mix was added and incubated for 45 min at 37°C in the dark, including blank wells (with non-stained cells). The fluorescence intensity was measured using a fluorescence plate reader (EnSpire™ 2300 Multilabel Reader; Perkin Elmer, Inc., Waltham, MA, USA) at excitation/emission=488/525 nm.

### Measurement of adenosine triphosphate (ATP) production

Intracellular ATP levels were measured using the ApoSENSOR cell viability assay kit (BioVision) according to the manufacturer’s instructions. Briefly, cells were treated with DHM (10, 50 and 100 μM) for 48 h, then incubated with 100 μl nuclear releasing reagent for 5 min at room temperature with gentle shaking, followed by further incubation with 5 μl ATP monitoring enzyme. Detection was performed using a luminometer (Sirius L; Titertek-Berthold, Pforzheim, Germany).

### Annexin V/propidium iodide (PI) double staining assay

Apoptotic cells were quantified using an Annexin V-fluorescein isothiocyanate (FITC)/PI kit (BioVision) and detected by flow cytometry (FACSCalibur; Becton-Dickinson, BD Biosciences, Franklin Lakes, NJ, USA), and analyzed by Modfit and CellQuest Vida 6.1 software (BD Biosciences). Briefly, cells were pretreated with 10, 50 or 100 μM DHM for 48 h and washed with phosphate-buffered saline (PBS). Cells were subsequently collected and resuspended in binding buffer [pH 7.5, 10 mM 4-(2-hydroxyethyl)-1-piperazineethanesulfonic acid, 2.5 mM CaCl_2_ and 140 mM NaCl]. Cells were incubated with Annexin V-FITC and PI for 10 min in the dark, prior to flow cytometric analysis. In the early stages of apoptosis, cells were Annexin V-positive, whereas Annexin V and PI-positive cells were considered to be in the late stage of apoptosis.

### MTT assay

Cell densities were adjusted to 2×10^4^ cells/100 μl. Cells were seeded into a 96-well plate, which was placed in an incubator overnight to allow for attachment and recovery. Briefly, cells were pretreated with 10, 50 or 100 μM DHM for 48 h. MTT was dissolved at 5 mg/ml in warm assay medium and 20 μl MTT solution was transferred to each well to yield a final volume of 120 μl/well. Plates were incubated for 4 h at 37°C and 5% CO_2_. Following incubation, supernatants were removed, and 150 μl DMSO was added. The plate was placed on an orbital shaker for 5 min and subsequently, the absorbance at 595 nm was recorded with an EnSpire™ 2300 Multilabel Reader (Perkin Elmer, Inc.).

### DHM-regulated protein analysis

Cells were collected following DHM treatment and lysed in lysis buffer [100 mM Tris-HCl, pH 6.8, 4% (m/v) SDS, 20% (v/v) glycerol, 200 mM 2-mercaptoethanol, 1 mM phenylmethyl sulfonylfluoride, and 1 g/ml aprotinin] for 30 min on ice. The total protein concentrations in the supernatants were detected using a bicinchoninic acid (BCA) assay with the BCA Protein Assay kit purchased from Beyotime Institute of Biotechnology (Haimen, Jiangsu, China). SDS-PAGE was performed using an 8–15% gradient on standard polyacrylamide gels. Proteins were subsequently transferred to nitrocellulose membranes saturated with 5% milk in Tris-buffered saline and 1% Tween-20 (TBST) and incubated with primary antibodies in diluent overnight at 4°C. The membranes were washed three times with TBST, incubated with anti-rabbit IgG-HRP for 1 h and washed a further four times with TBST. Detection was performed using the Odyssey Infrared Imaging System (LI-COR Biosciences Inc., Lincoln, NE, USA).

### Quantitative PCR (qPCR): Quantification of messenger RNA (mRNA) expression

mRNA expression levels were determined by qPCR using SYBR green. mRNA was reverse-transcribed to cDNA using the PrimeScript RT Reagent kit with the gDNA Eraser kit (Takara Bio, Inc., Otsu, Japan) The following primer sequences were used: 18S forward, 5′-CGGCGACGACCCATTCGAAC-3′ and reverse, 5′-GAATCGAACCCTGATTCCCCGTC-3′; TGF-β forward, 5′-GGACTACTATGCTAAAGAGGTCAC-3′ and reverse, 5′-CTGTATTCCGTCTCCTTGGTTCAGC-3′; NOX4 forward, 5′-GTTCGGCACATGGGTAAAAG-3′ and reverse, 5′-ACCAAGGGCCAGAGTATCAC-3′. Total RNA was prepared using TRIzol reagent (Invitrogen Life Technologies, Carlsbad, CA, USA). qPCR was performed with the MJ chromo 4 RT-PCR detection system (Bio-Rad Laboratories, Hercules, CA, USA). The expression levels of the housekeeping gene 18S were measured as an internal control.

### Statistical analysis

All values are presented as the mean ± standard deviation from triplicate experiments performed in a parallel manner unless otherwise indicated. Statistical differences were evaluated using Student’s t-test. P<0.05 was considered to indicate a statistically significant difference between values. All figures exhibited in the present study are representative of ≥three independent experiments.

## Results

### DHM inhibits proliferation and promotes apoptosis of Hepal-6 cells

**Figure 1:**
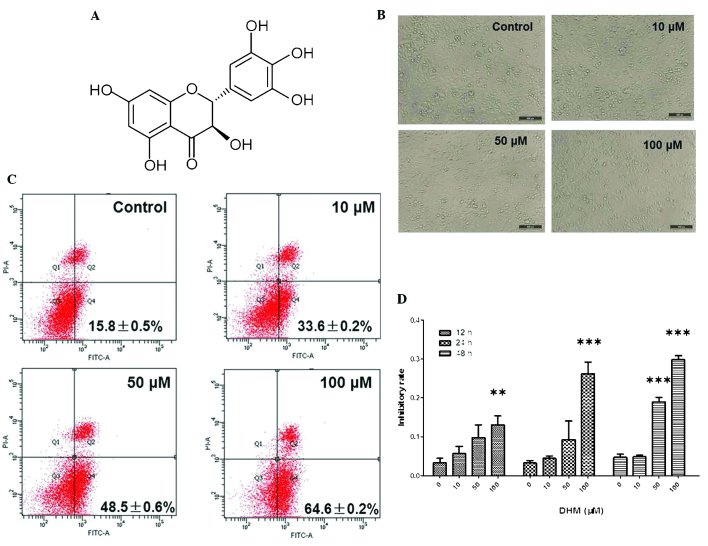
DHM induces cell growth inhibition and apoptosis in Hepal-6 cells. (A) Chemical structure of DHM. (B) DHM induced cell proliferation in Hepal-6 at various concentrations (10, 50 and 100 μM) for 48 h, visualized by microscopy (magnification, ×100). (C) Hepal-6 cells were treated with various concentrations (10, 50, or 100 μM ) of DHM for 48 h and the results were analyzed by flow cytometry. Each sample was measured in duplicate, and the figure is a representative of three independent assays. (D) MTT assay analyzed cell growth inhibition rates in cells treated with different concentrations (10, 50 and 100 μM) of DHM for 12, 24, 48 h. Values are expressed as the mean ± standard deviation of three independent experiments. ^**^P<0.01, ^***^P<0.001 vs. 0 μM DHM. DHM, dihydromyricetin; FITC, fluorescein isothiocyanate; PI, propidium iodide; A, area.

Untreated Hepal-6 cells grew normally with clear skeletons, whereas the morphology of cells treated with DHM was distorted, some became round and the number of sloughed cells increased in a dose-dependent manner (Fig. 1B). The rate of cell apoptosis also increased in a concentration-dependent manner (Fig. 1C). The results of the MTT assay demonstrated that DHM inhibited cell growth in a time- and concentration-dependent manner in Hepal-6 cells following 12, 24 and 48 h treatment (Fig. 1D). These data revealed that DHM exerted a significant inhibitory effect on the viability of Hepal-6 cells, which may contribute to its anti-tumor potency. In cells treated with 50 μM DHM cell growth was inhibited and the majority of Hepal-6 cells underwent apotosis (IC_50_ of DHM on Hepal-6 cells was 190 μM for 48 h treatment). These results demonstrated that DHM inhibited proliferation and promoted apoptosis in Hepal-6 cells in a time- and concentration-dependent manner.

### DHM reduces ROS production in Hepal-6 cells

**Figure 2:**
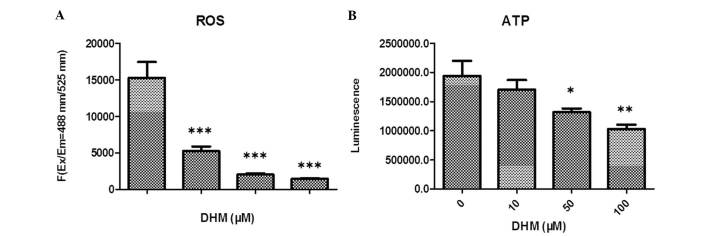
DHM decreases ROS and ATP in Hepal-6 cells. (A) Intracellular levels of ROS were detected following 48 h treatment with DHM (10, 50 and 100 μM). (B) Intracellular levels of ATP were detected following 48 h treatment with DHM (10, 50 and 100 μM). ^*^P<0.05; ^**^P<0.01; ^***^P<0.001 vs. 0 μM DHM. DHM, dihydromyricetin; ROS, reactive oxygen species; ATP, adenosine triphosphate.

The levels of ROS in Hepal-6 cells treated with various concentrations of DHM for 48 h were evaluated. The cell-permeant DCFDA, which is oxidized to green fluorescent 2′,7′-dichlorofluorescein by various peroxide-like ROS and nitric oxide-derived reactive intermediates, was used as a probe. These data demonstrated that DHM significantly decreased ROS production in Hepal-6 cells, and that this ROS imbalance may promote mitochondrial dysfunction and trigger mitochondria-mediated apoptosis. Intracellular levels of ROS in cells treated with 10, 50 and 100 μM DHM decreased in a concentration-dependent manner, compared with those in vehicle-treated cells (Fig. 2A).

### DHM decreases intracellular ATP expression levels in Hepal-6 cells

In order to examine whether DHM caused a dysfunction of mitochondrial energy, intracellular levels of ATP in DHM-treated cells were investigated. Cells were treated with various concentrations of DHM for 48 h and the results indicated that the intracellular levels of ATP were markedly decreased in a concentration-dependent manner (Fig. 2B).

### DHM downregulates TGF-β and NOX4

**Figure 3:**
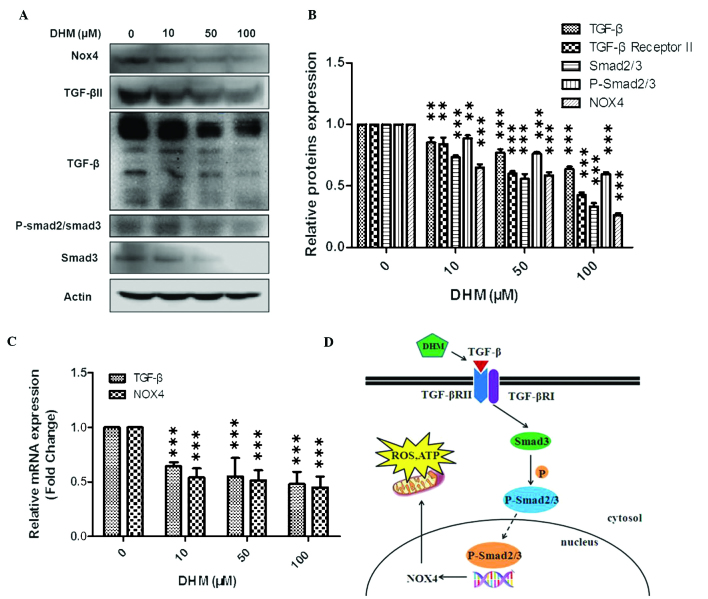
Western blot analysis of the effects of DHM on mRNA and protein expression levels in Hepal-6 cells. (A) Western blot analysis of cells treated with various concentrations (10, 50 and 100 μM) of DHM for 48 h (representative of three independent experiments). (B) Column diagram for A. (C) Cells were treated with DHM (10, 50 and 100 μM) for 24 h and TGF-β and NOX4 mRNA expression were measured by quantitative polymerase chain reaction. The experiment was performed in triplicate. ^**^P<0.01, ^***^P<0.001 vs. 0 μM DHM. Values are expressed as the mean ± standard deviation (D) Schematic of the suggested mechanism of action of DHM. DHM, dihydromyricetin; mRNA, messenger RNA; NOX4, NADPH oxidase 4; TGF-β, transforming growth factor-β; ROS, reactive oxygen species; ATP, adenosine triphosphate; TGF-βR, transforming growth factor-β receptor; p, phosphorylated.

In the present study, cells were treated with 10, 50 or 100 μM DHM for 24 h and mRNA expression levels of TGF-β and NOX4 were evaluated by qPCR. Cells were also treated with 10, 50 or 100 μM DHM for 48 h and TGF-β, TGF-β II, Smad3, p-Smad2/3 and NOX4 protein expression levels were evaluated by western blot analysis. The results indicated that TGF-β and NOX4 mRNA expression levels decreased, and that protein expression levels of TGF-β, TGF-β II, Smad3, p-Smad2/3 and NOX4 were reduced in a concentration-dependent manner (Fig. 3A–C).

## Discussion

Western blot analysis was performed in order to measure TGF-β, TGF-βRII, Smad3, p-Smad2/3 and NOX4 protein expression levels, while qPCR analysis was used to measure TGF-β and NOX4 mRNA expression levels. The results demonstrated that DHM decreased TGF-β and NOX4 mRNA expression levels in cells treated with DHM for 24 h and furthermore, induced a reduction in TGF-β, TGF-βRII, Smad3, p-Smad2/3 and NOX4 protein expression levels in a concentration-dependent manner. It was further demonstrated that DHM induced a decrease in ROS and ATP production in a concentration-dependent manner. The TGF-β signaling pathway is involved in multiple cellular processes, including cell growth, differentiation, adhesion, migration and apoptosis. TβRI, TβRII and intracellular mediators, including Smad proteins, mediate TGF-β signaling (22,23). The binding of TGF-β to TβRII induces phosphorylation of TβRI at glycine-serine repeats in the cytoplasmic tail domain by TβRII, leading to TβRI activation (24). At present, it is hypothesized that the Smad complex remains associated and is actively involved in transcriptional regulation (25,26). A previous study demonstrated that Smad3 and -4 had important roles in TGF-β-induced epithelial to mesenchymal transition and breast cancer metastasis (27). It was reported that abrogation of the Smad pathway in M4 cells by using a dominant negative Smad3 mutant or via overexpression of a Smad-binding defective TβRI mutant suppressed metastasis (28,29).

Cancer progression has been associated with oxidative stress (30). Loss of TGF-β signaling in mammary carcinoma cells increased the abundance of smooth muscle actin-positive stroma and enhanced tumor cell survival and heterogeneity (31–33). A study by Giannelli *et al* (34) indicated that the inhibition of TGF-β signalling resulted in numerous downstream effects, which may improve clinical outcomes in hepatocellular carcinoma treatment. Furthermore, it has been demonstrated that ROS production in human hepatocyte cell lines previously infected with the hepatitis C virus depends on NOX4 activity, whose expression is stimulated by TGF-β (18). Superoxide and hydrogen peroxide, which are redox signaling molecules involved in various cellular functions, are major producers of ROS (35). Redox imbalance occurs due to excessive or insufficient ROS production and is a pathophysiological induction factor for numerous pathological conditions, including cancer development and progression. It has previously been demonstrated that, apart from mitochondria, the nicotinamide adenine dinucleotide phosphate oxidase complex is the most significant intracellular source of ROS (36). NOX4 expression has been demonstrated to be regulated by differentiating factors including TGF-β, as observed in the present study (37). It was also reported that TGF-β1-stimulated expression of NOX4 resulted in the oxidation of mitogen-activated protein kinase phosphatase-1, which led to the factor-dependent modification of gene expression in murine fibroblasts (38). Several studies have reported that TGF-β induces NOX4 to generate intracellular ROS (19,39). Smad3-mediated gene transcription has an important role in the induction of NOX4 expression following TGF-β stimulation (40).

In conclusion, the present study revealed that DHM induced a reduction in TGF-β, TGF-βRII, Smad3, p-Smad2/3 and NOX4 protein expression levels, as well as a reduction in ROS and ATP production in Hepal-6 cells. Conventional anti-cancer drugs induce cancer cell apoptosis by elevating ROS; however this results in significant damage to normal cells. DHM enhanced the rate of apoptosis in Hepal-6 cells, whilst reducing ROS levels. This means that DHM may be capable of exerting anti-cancer effects whilst causing minimal damage to normal cells. Further studies are required to elucidate the potential of DHM to be used as an anti-cancer drug.
